# Video analysis of *ex vivo* beating hearts during preservation on the TransMedics® organ care system

**DOI:** 10.3389/fcvm.2023.1216917

**Published:** 2023-06-20

**Authors:** Michelle Mendiola Pla, Silvia Berrettoni, Franklin H. Lee, Giacomo Rozzi, Federica Marrano, Ryan T. Gross, Amy Evans, David C. Wendell, Paul Lezberg, Margherita Burattini, Francesco Paolo lo Muzio, Lorenzo Fassina, Carmelo A. Milano, Marie-Louise Bang, Dawn E. Bowles, Michele Miragoli

**Affiliations:** ^1^ Department of Surgery, Duke University Medical Center, Durham, NC, United States; ^2^ Department of Medicine and Surgery, University of Parma, Parma, Italy; ^3^ Perfusion Services, Duke University Medical Center, Durham, NC, United States; ^4^ Duke Cardiovascular Magnetic Resonance Center, Duke University Medical Center, Durham, NC, United States; ^5^ TransMedics, Inc., Andover, MA, United States; ^6^ Department of Surgical Sciences, Dentistry, and Maternity, University of Verona, Verona, Italy; ^7^ Department of Electrical, Computer, and Biomedical Engineering, University of Pavia, Pavia, Italy; ^8^ Institute of Genetic and Biomedical Research (IRGB), National Research Council (CNR), Milan Unit, Milan, Italy; ^9^ IRCCS Humanitas Research Hospital, Milan, Italy

**Keywords:** *ex vivo* perfusion, normothermic, video, kinematics, biomarker, cardiac transplantation

## Abstract

**Background:**

Reliable biomarkers for assessing the viability of the donor hearts undergoing *ex vivo* perfusion remain elusive. A unique feature of normothermic *ex vivo* perfusion on the TransMedics® Organ Care System (OCS™) is that the donor heart is maintained in a beating state throughout the preservation period. We applied a video algorithm for an *in vivo* assessment of cardiac kinematics, video kinematic evaluation (Vi.Ki.E.), to the donor hearts undergoing *ex vivo* perfusion on the OCS™ to assess the feasibility of applying this algorithm in this setting.

**Methods:**

Healthy donor porcine hearts (*n* = 6) were procured from Yucatan pigs and underwent 2 h of normothermic *ex vivo* perfusion on the OCS™ device. During the preservation period, serial high-resolution videos were captured at 30 frames per second. Using Vi.Ki.E., we assessed the force, energy, contractility, and trajectory parameters of each heart.

**Results:**

There were no significant changes in any of the measured parameters of the heart on the OCS™ device over time as judged by linear regression analysis. Importantly, there were no significant changes in contractility during the duration of the preservation period (time 0–30 min, 918 ± 430 px/s; time 31–60 min, 1,386 ± 603 px/s; time 61–90 min, 1,299 ± 617 px/s; time 91–120 min, 1,535 ± 728 px/s). Similarly, there were no significant changes in the force, energy, or trajectory parameters. Post-transplantation echocardiograms demonstrated robust contractility of each allograft.

**Conclusion:**

Vi.Ki.E. assessment of the donor hearts undergoing *ex vivo* perfusion is feasible on the TransMedics OCS™, and we observed that the donor hearts maintain steady kinematic measurements throughout the duration.

## Introduction

*Ex vivo* machine perfusion has transformed organ transplantation outcomes by minimizing ischemic injury and reconditioning the organs prior to transplantation (1–3). Its use in clinical practice continues to grow as it has permitted for longer preservation times and for the utilization of the organs that would have traditionally been excluded from transplantation. In cardiac transplantation, normothermic *ex vivo* perfusion (NEVP) has allowed for the expansion of the donor pool through the utilization of hearts from donors after circulatory death (4). However, reliable measures for assessing the functionality and health quality of the donor heart remains elusive.

Currently, lactate measured in the perfusate is viewed by many as a proxy for injury and stress of the donor heart over time. Despite this, it has been well described that lactate is a poor predictor of post-operative graft outcomes (5, 6). A unique feature of NEVP is that the donor heart is maintained in a beating state throughout the preservation period and can be directly observed to assess the quality of the donor organ (7). Cardiac transplant surgeons can qualitatively assess the contractility of a donor heart as a parameter to determine its fitness for transplantation.

We applied a well-characterized video method for *in vivo* assessment of cardiac kinematics called video kinematic evaluation (Vi.Ki.E.) (8–10) and assessed the feasibility of using this method to measure *ex vivo* cardiac kinematics while a porcine donor heart is undergoing NEVP on the TransMedics® Organ Care System (OCS™). A successful measurement of cardiac kinematics while the donor hearts are beating on the OCS™ could allow for this technology to be applied as a biomarker to predict cardiac fitness.

## Methods

### Donor heart procurement and *ex vivo* perfusion

This study was approved by the Duke University Institutional Animal Care and Use Committee. Female Yucatan pigs (Sinclair Bio Resources, Auxvasse, MO, United States) aged 7–9 months were utilized for this study. Baseline cardiac troponin I values and cardiac magnetic resonance imaging (cMRI) were obtained prior to surgery. In preparation for surgery, the animals were anesthetized and intubated for mechanical ventilation. The donor hearts (*n* = 6) were procured in a standard fashion through a sternotomy. The hearts were then prepared on a back table and subsequently mounted on an OCS™ and underwent 2 h of NEVP at 34°C–35°C as described by Mendiola Pla et al. (11). During this time, the perfusion parameters of aortic flow, aortic pressure, heart rate, perfusion temperature, venous oxygen saturation (SvO_2_), perfusate lactate, and perfusate hematocrit (Hct) were obtained.

### Video acquisition

**Figure 1:**
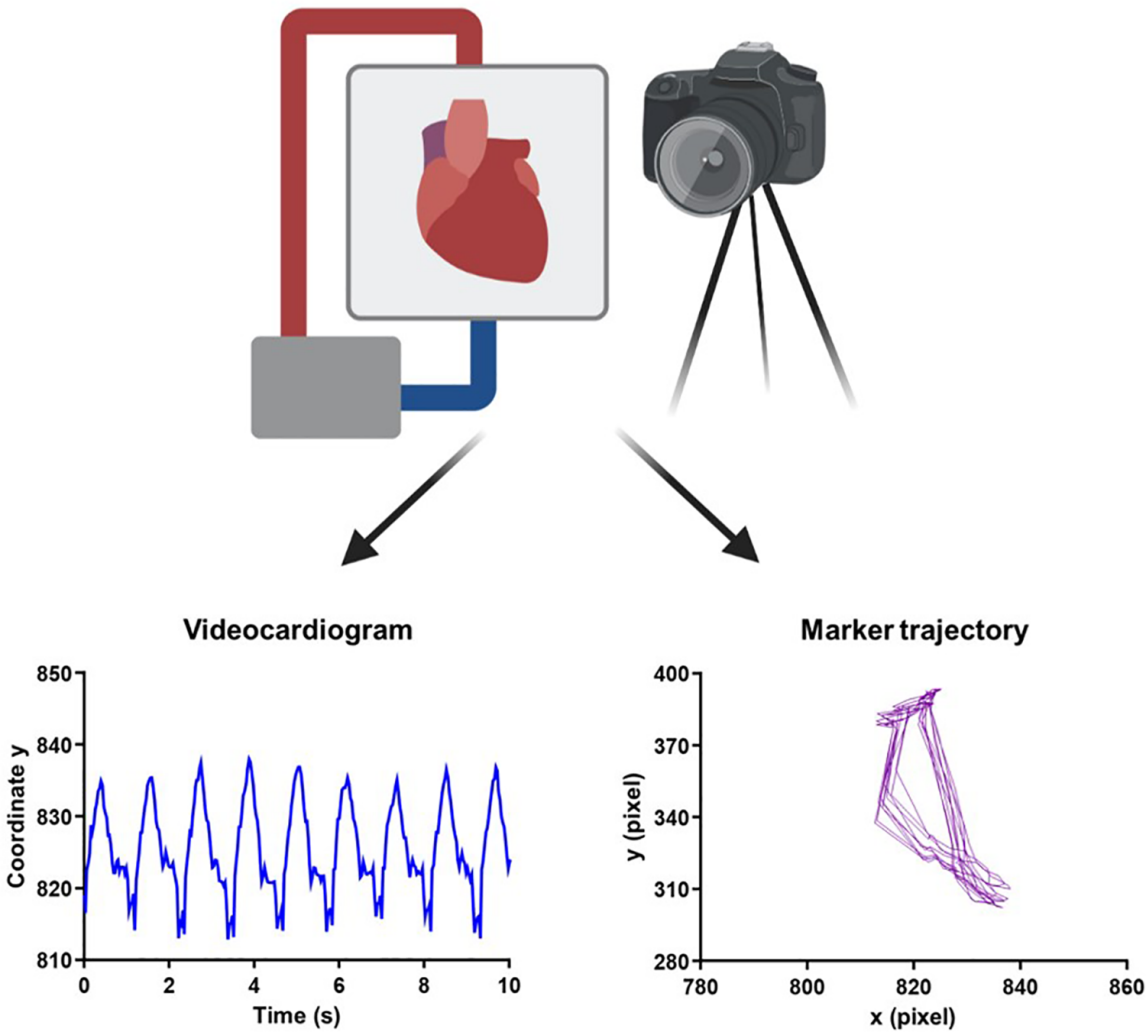
Experimental overview of video acquisition and kinematic analysis. Schematic representation of the camera positioned in front of the heart while it is undergoing normothermic *ex vivo* perfusion on the OCS. Using a Vi.Ki.E.-customized software, the trajectory of contraction (left to right) and relaxation (right to left) for every cardiac cycle was traced. A ViCG showing the displacement of a video marker with contraction/relaxation peaks and intervals among consecutive peaks was also traced. The schematic representation was created on BioRender. OCG, Organ Care System; Vi.Ki.E., video kinematic evaluation; ViCG, video cardiogram.

Video recordings were obtained using either a Nikon D5600 equipped with a Nikon 18–55 mm f/3.5–5.6G VR lens (Nikon Inc., Melville, NY, United States) or a Canon EOS Rebel T8i equipped with a Canon EFS 18–55 mm lens (Canon, Inc., Melville, NY, United States). Once perfusion of the donor heart was established on the OCS™ and the heart was beating, the camera was positioned approximately 30–40 cm perpendicularly in front of the heart (Figure 1). The distance between the camera and the heart, the focus, lighting, and orientation of the heart remained unchanged during and between recordings once these parameters were established. Serial high-resolution videos were recorded every 15 min during the perfusion period at a recording frequency of 30 frames per second (fps). All hearts were in normal sinus rhythm during the recordings.

### Heterotopic heart transplantation and follow-up

Following the *ex vivo* perfusion and video acquisition, the hearts were cooled to 14°C–16°C, then arrested and removed from the OCS™. The heart was then prepared for transplantation in a standard fashion and transplanted into the recipient pig in an intra-abdominal position with the graft aorta anastomosed to the recipient aorta and the graft pulmonary artery anastomosed to the recipient inferior vena cava (11). Echocardiographic assessments of each transplanted heart were obtained between 2–6 post-operative days.

### Quantitative analysis

**Figure 2:**
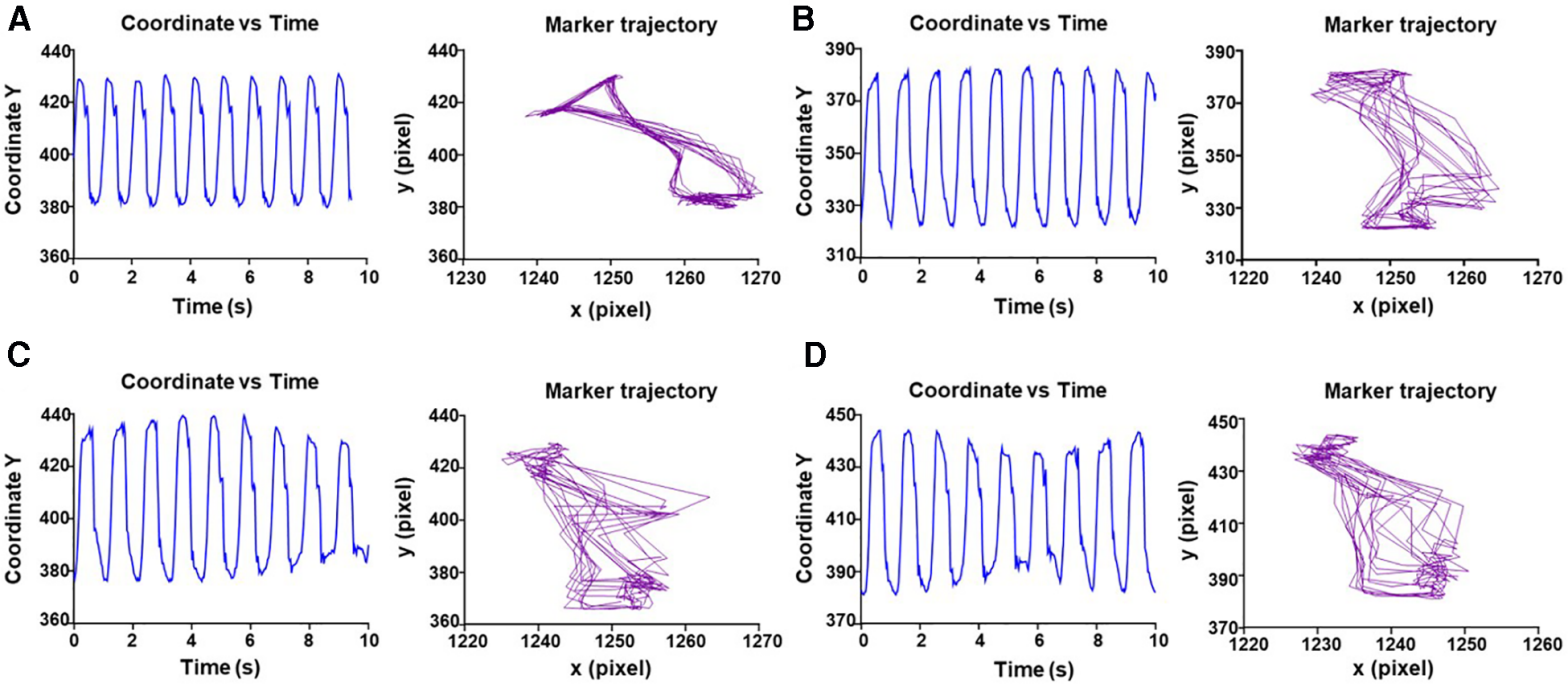
Representative evaluation of trajectory and displacement of marker 1 over time. (**A**) Displacement of video marker 1 with contraction/relaxation peaks and trajectory of contraction (left to right) and relaxation (right to left) for every cardiac cycle at 0 min. (**B–D**) Same as **A** for 30–60–105 min, respectively.

Cardiac performance on the OCS™ was evaluated using Vi.Ki.E. by extraction of the kinematic parameters every 15 min during spontaneous beating, while monitoring the heart for 2 h (Figure 1). As shown in Supplementary Video S1, a virtual marker was placed on top of the beating heart and followed using a video spot tracker (VST), an open-software (https://cismm.web.unc.edu/resources/software-manuals/video-spot-tracker-manual/) capable of returning the XY coordinates of the marker movement for every cardiac beat. The selected VST kernel followed the heart movement and created the trajectory of contraction and relaxation (Figure 2) in the XY plane.

The coordinates are then analyzed using the Vi.Ki.E. system, which is written in MATLAB programming language (MathWorks, Inc., Natick, MA, United States) and returns the kinematic parameters such as contractility (maximal contraction velocity), cardiac force, energy expenditure, and trajectory perimeter (tissue compliance), as previously described by Rozzi et al. (10).

**Figure 3:**
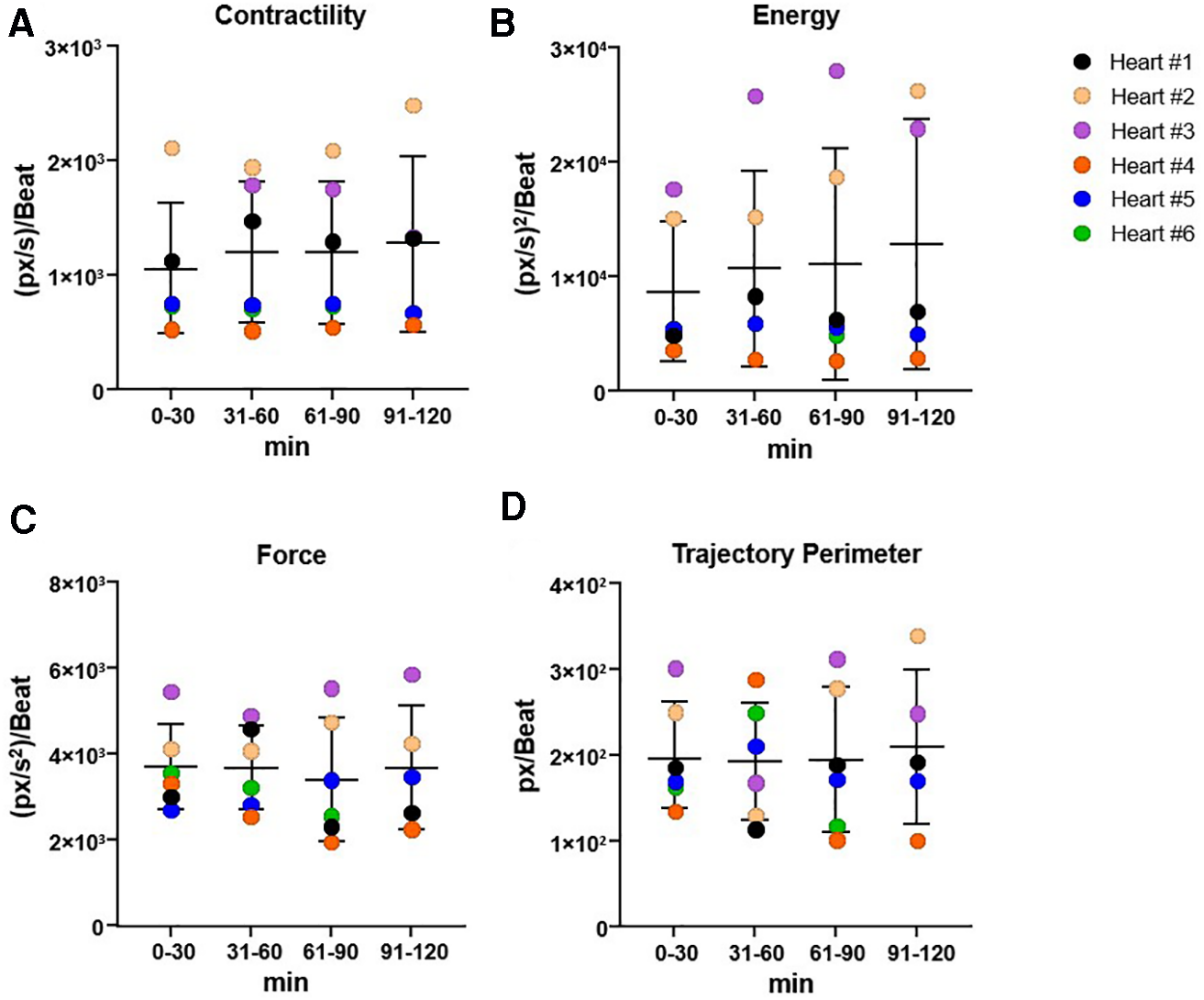
Overview of cardiac kinematic parameters over time. Cardiac kinematic parameters, such as cardiac fatigue (**A**), energy (**B**), contractility (**C**), and trajectory perimeter (**D**), grouped in range of 30 min, to evaluate the trend over time. The data used were obtained from a high-resolution video acquired every 15 min for 2 h, and analyzed with Vi.Ki.E. Data expressed as mean ± SD, using one-way ANOVA (significance set at *P* < 0.05). Vi.Ki.E., video kinematic evaluation.

To investigate whether the kinematic parameters such as contractility (expressed as maximal contraction velocity), contraction force (indicating cardiac fatigue), energy (expenditure of energy during contraction/relaxation), and trajectory perimeter (indication of cardiac compliance) were modified over time, data acquisition was divided into four temporal windows (0–30, 31–60, 61–90, and 91–120 min) (Figure 3).

### Statistics

Data are expressed as mean ± SD. Normality was assessed by the Kolmogorov–Smirnov test. Comparisons were performed using one-way ANOVA with Bonferroni *post-hoc* test for multiple comparisons. Statistical analyses were performed using GraphPad Prism version 9.5.1 (GraphPad Software, San Diego, CA, United States). The details of the specific test used for each experiment are reported in the figure legends. *P*-values <0.05 were considered statistically significant.

## Results

### Pre-operative donor heart function and perfusion parameters

The baseline cardiac MRI demonstrated no evidence of compromised function in any of the donor hearts with the ejection fraction (EF) measuring >50%. The representative MRI data are shown in Supplementary Figure S1 and Supplementary Video S2. The baseline troponin levels for each pig are shown in Supplementary Table S1. The median cardiac troponin I value was 22 ng/L with an interquartile range from 19 to 31 ng/L. Cardiac troponin I was elevated in only one of the pigs (865 ng/L) for unknown reasons. However, on gross inspection of the heart at the time of sternotomy, there was no evidence of cardiac injury or compromised activity.

The composition of OCS™ perfusate is shown in Supplementary Table S2. It is donor blood-based and supplemented with several additives to maintain near physiologic function of the donor heart throughout the perfusion period. Supplementary Figure S2 demonstrates the OCS™ perfusion parameters measured throughout the perfusion period. The parameters were largely consistent between each of the donor hearts: average aortic flow, 0.61–0.72 L/min; average aortic pressure, 56.2–65.6 mmHg; average heart rate, 56–105 bpm; temperature, 33.9°C; SvO_2_, 88.4%–96.0%; and Hct, 18.7%–28.4%. The average total perfusion time was 140 min with a standard deviation of 18 min. Supplementary Figure S3 shows the plotted lactate trends of each heart during perfusion on the OCS, with minimal differences noted between each heart and each remaining within normal limits (<1.5 mmol/L).

### Video kinematic parameters

While monitoring the heart for 2 h, we did not observe changes in spontaneous beating frequency (Figure 2). This may be attributed to the accommodation of the heart to the new environment. Despite the wide distribution of the data, likely due to differences between each of the hearts, we did not observe significant changes in both contractility and energy parameters over time in any of the hearts. This kinematic parameter ranged from 918.0 ± 430 px/s at the start of perfusion to 1,535 ± 728.5 px/s at the end of perfusion (Figure 3A). Force measurements ranged from 3,776 ± 1,357 (px/s^2^)/beat at the start of perfusion to 3,350 ± 897.4 (px/s^2^)/beat at the end of perfusion (Figure 3B). Energy measurements ranged from 6,274 ± 3,240 (px/s)^2^/beat at the start of perfusion to 16,948 ± 11,262 (px/s)^2^/beat at the end of perfusion (Figure 3C). Finally, the trajectory perimeter measurements ranged from 199.3 ± 72.52 px/beat at the start of perfusion to 241.7 ± 83.59 px/beat at the end of perfusion (Figure 3D).

**Figure 4:**
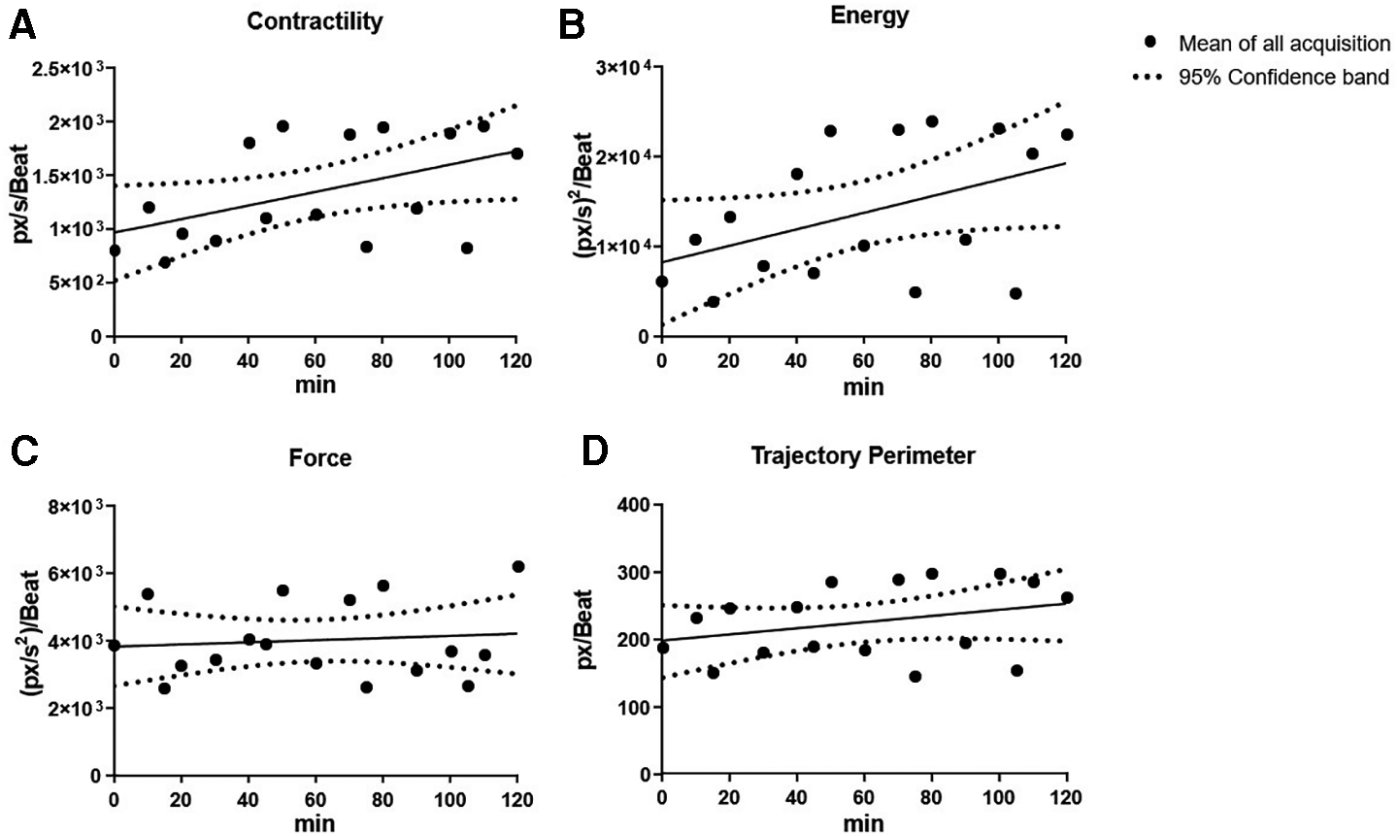
Overview of cardiac kinematics parameters over time with linear regression. Cardiac kinematic parameters [cardiac fatigue (**A**), energy (**B**), contractility (**C**), and trajectory perimeter (**D**)] were assessed over time using linear regression. Contractility and energy showed a slightly increasing slope, while force and trajectory perimeter had a nearly flat regression over time suggesting that cardiac fatigue and tissue compliance remained constant over time.

This was further assessed and confirmed by linear regression analysis (Figure 4). Contractility and energy showed a slightly increasing slope in the regression lines *y* = 6.260 × *x* + 969.8 and *y* = 91.49 × *x* + 8,296, respectively. On the other hand, force and trajectory perimeter exhibited a nearly flat regression over time in the regression lines *y* = 2.937 × *x* + 3,844 and *y* = 0.4509 × *x* + 198.3, suggesting that cardiac fatigue and tissue compliance remained constant over the period of the experiment.

### Post-operative donor heart function

Each heart demonstrated robust biventricular contractility on post-operative echocardiography, which were all consistently performed by MMP. A representative recording is shown in Supplementary Video S3.

## Discussion

We present the first report describing the utility of the Vi.Ki.E. system to assess the kinematics of *ex vivo* beating hearts undergoing normothermic perfusion on the TransMedics OCS™. This technology could potentially be utilized to provide quantitative assessments of cardiac fitness for the hearts preserved on the OCS™ that could aid surgeons to decide whether a donor heart is suitable for transplantation. There are currently no reliable quantitative measures to assess donor heart fitness prior to transplantation. The utility of such a measure is important to be able to medically prepare for or even prevent outcomes of moderate or severe primary graft dysfunction (PGD). This is of great value since moderate PGD is associated with a 12% risk of mortality or re-transplantation and severe PGD with a 40%–50% risk (12). Lactate measured from the perfusate is the most used biomarker; however, it has been shown to correlate modestly with post-transplantation outcomes.

In this study, we chose to investigate four kinematic parameters that are essential for evaluating cardiac kinematic function: contractility, force, energy, and trajectory perimeter. Contractility refers to the maximal contraction velocity of the heart muscle, while force is an indication of cardiac fatigue. Energy represents the expenditure of energy during contraction and relaxation, and trajectory perimeter is an indicator of cardiac compliance. By monitoring these parameters over time, we aimed to determine if the performance of the heart changes during the *ex vivo* preservation time. To this end, we utilized healthy donor hearts to perform these studies. The results showed that there were no significant changes in any of the kinematic parameters over time. This suggests that the function and performance of the heart remain stable throughout the *ex vivo* preservation period on the TransMedics OCS™. Following the preservation period, we demonstrated that the cardiac allografts maintained robust contractility on post-transplantation echocardiography.

Given the ability to analyze cardiac fitness prior to transplantation in a non-invasive manner, future studies are warranted where Vi.Ki.E. is applied to analyze the kinematic parameters of human hearts undergoing NEVP on the OCS™ and correlated with post-transplantation outcomes. The association of the kinematic measures with clinical outcomes could be used to develop an artificial intelligence (AI) platform that can predict PGD outcomes in patients based on the beating activity of the donor heart on the OCS™. The application of Vi.Ki.E. to guide AI assessments of the heart has been previously described (13). Potential translation of this technology to clinical practice would help to reduce subjective clinical decision making when assessing the donor hearts for transplantation and provide a possible standardized measure.

In conclusion, our study provides valuable insights into the performance of *ex vivo* beating hearts on the OCS™ system using the Vi.Ki.E. system. The results suggest that the cardiac function and performance remain stable on the OCS™, which is an encouraging finding for the expansion of the utility of normothermic *ex vivo* perfusion for donor heart preservation during transplantation.

## Data Availability

The raw data supporting the conclusions of this article will be made available by the authors, without undue reservation.

## Supplementary material

Supplementary Figure S1 *Representative baseline cardiac magnetic resonance imaging (MRI) of a donor heart*. (A) Freeze frame of cine image during diastole (B) T1 mapping of cardiac MRI taken of donor heart prior to transplantation.

Supplementary Figure S2 *Summary of measured OCS perfusion parameters*.

Supplementary Figure S3 *Plotted lactate trends measured from the OCS perfusate*.

Supplementary Video S1 *Representative video of virtual marker tracing of a beating heart using Video Spot Tracker*.

Supplementary Video S2 *Representative baseline MRI cine of donor heart before transplantation*.

Supplementary Video S3 *Representative post-operative echocardiogram of allograft post-transplantation*.
