# Supplementary figures and images for: Video analysis of ex vivo beating hearts during preservation on the TransMedics® organ care system
Source: Front Cardiovasc Med. 2023 Jun 20;10:1216917. doi: 10.3389/fcvm.2023.1216917 (PMC10318359; doi:10.3389/fcvm.2023.1216917)

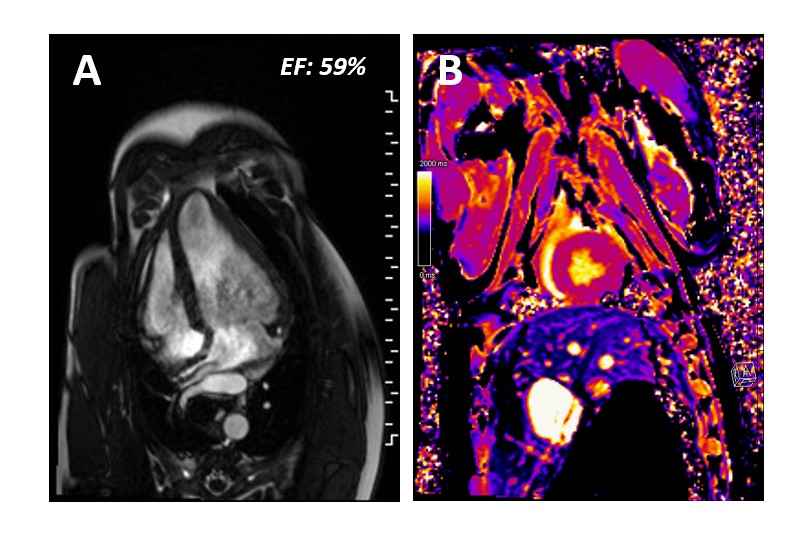

Supplement: Supplementary Figure S1 — Representative baseline cardiac magnetic resonance imaging (MRI) of a donor heart. (A) Freeze frame of cine image during diastole (B) T1 mapping of cardiac MRI taken of donor heart prior to transplantation. [file Image1.jpeg]

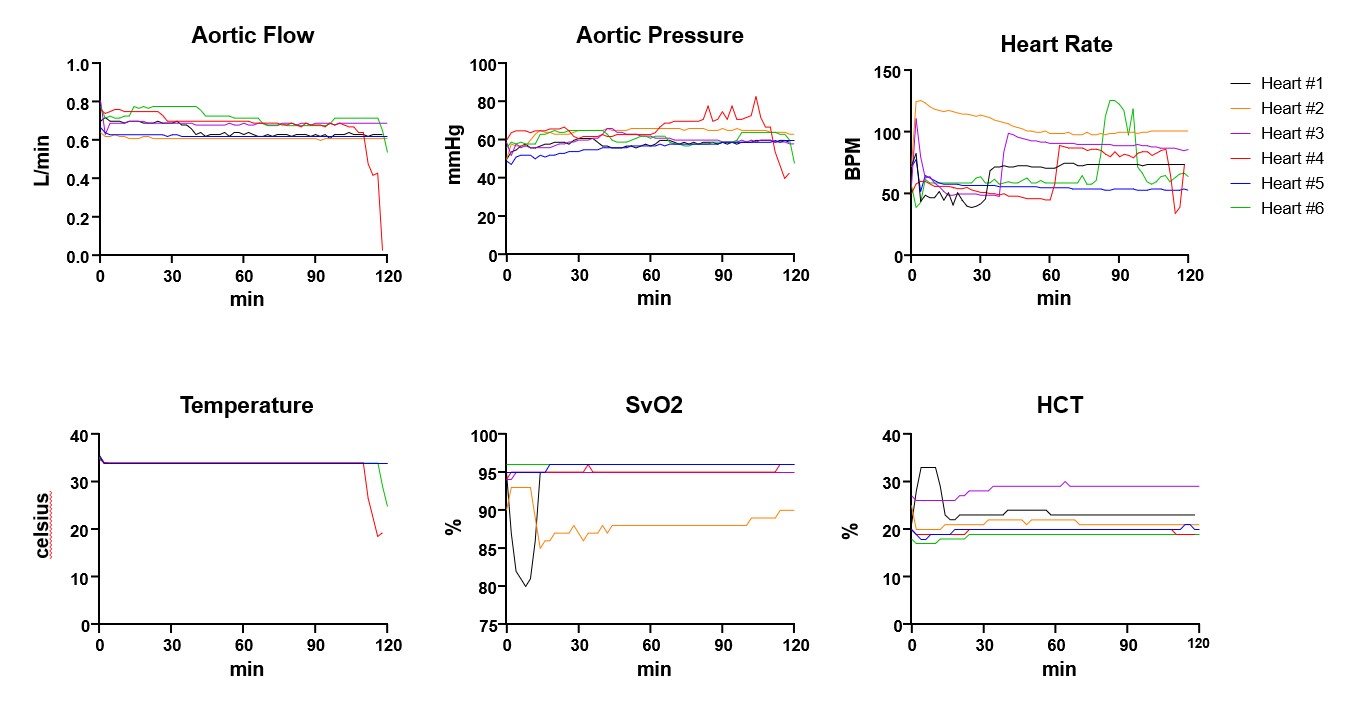

Supplement: Supplementary Figure S2 — Summary of measured OCS perfusion parameters. [file Image2.jpeg]

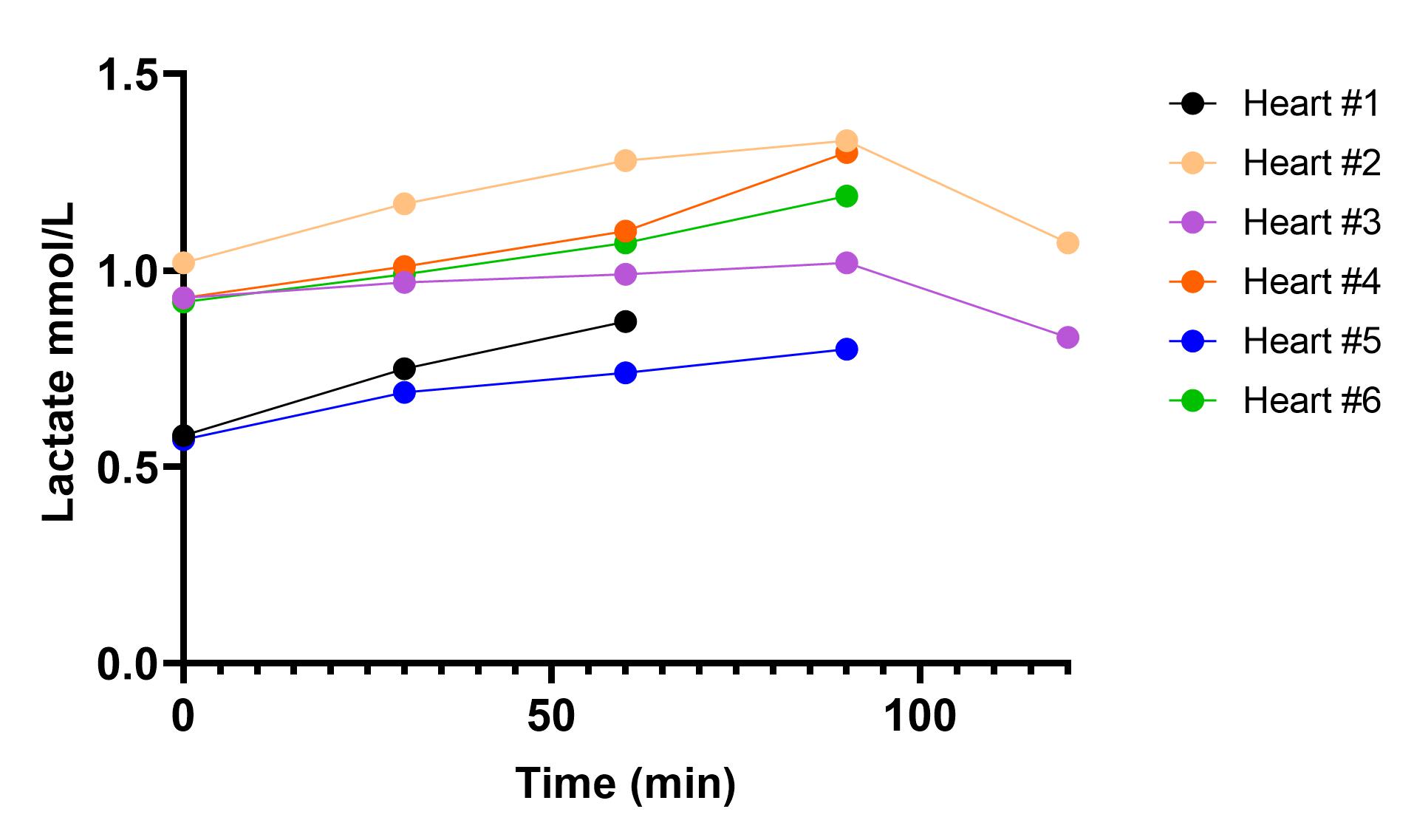

Supplement: Supplementary Figure S3 — Plotted lactate trends measured from the OCS perfusate. [file Image3.jpeg]
