# Supplementary material for: Video analysis of ex vivo beating hearts during preservation on the TransMedics® organ care system
Source: Front Cardiovasc Med. 2023 Jun 20;10:1216917. doi: 10.3389/fcvm.2023.1216917 (PMC10318359; doi:10.3389/fcvm.2023.1216917)
Supplement: Supplementary file 7 [file Table1.docx]

Supplementary Table 1: *Baseline donor pig troponin values.*

| **Donor Heart #** | **Troponin (ng/L)** |
| --- | --- |
| 1 | 4 |
| 2 | 22 |
| 3 | 19 |
| 4 | 865 |
| 5 | 31 |
| 6 | – |
| Median (IQR) | 22 (19, 31) |
