# Supplementary material for: Video analysis of ex vivo beating hearts during preservation on the TransMedics® organ care system
Source: Front Cardiovasc Med. 2023 Jun 20;10:1216917. doi: 10.3389/fcvm.2023.1216917 (PMC10318359; doi:10.3389/fcvm.2023.1216917)
Supplement: Supplementary file 8 [file Table2.docx]

Supplementary Table 2. *Composition of OCS perfusate.*

| **Perfusate Composition** | **Volume** |
| --- | --- |
| **Circuit Prime** |  |
| TransMedics Priming Solution | 500mL |
| 25% Albumin 12.5g/50mL | 100mL |
| Ciprofloxacin in D5W 200mg/100mL | 50mL |
| Cefazolin sodium 1g | 5mL |
| Multi-vitamin injection | 10mL |
| Methylprednisolone 125mg/2mL | 4mL |
| Sodium bicarbonate 50mEq/50mL | 20mL |
| Heparin 10,000U/10mL | 10mL |
| **Blood collection and Reconstitution** |  |
| Donor blood post wash | *Variable* |
| 5% Albumin 12.5g/250mL | *Variable* |

**Calcium gluconate, sodium bicarbonate, and dextrose are titrated as needed*
